# The impact of brivaracetam on cognitive processes and anxiety in various experimental models

**DOI:** 10.1007/s43440-023-00564-3

**Published:** 2024-01-05

**Authors:** Ewa Zwierzyńska, Bogusława Pietrzak

**Affiliations:** Department of Pharmacodynamics, Medical University of Lodz, Muszyńskiego 1, 90-151 Łódź, Poland

**Keywords:** Anxiety, Cognitive processes, Brivaracetam, Memory, Rats

## Abstract

**Background:**

Memory deficits and anxiety symptoms are undesirable effects that occur in epilepsy patients. They may be associated with the pathophysiology of the disease but also with anticonvulsant therapy. Brivaracetam (BRV) is one of the newest antiseizure drugs. It acts as a ligand for synaptic vesicle glycoprotein 2A (SV2A), which may play a significant role in cognitive processes. Although BRV has a favorable safety profile, its central side effects remain unclear. Hence, this study aimed to evaluate the effect of BRV on various types of memory and anxiety in rats.

**Methods:**

BRV was given to adult male Wistar rats (*n* = 80) via gastric tube as a single dose (6 mg/kg or 20 mg/kg) or chronically (6 mg/kg). The effect of the drug on spatial memory was evaluated in the Morris water maze (MWM), fear-learning by passive avoidance (PA), and recognition memory with novel object recognition (NOR). The elevated plus maze (EPM) was used to assess anxiety-like behaviors.

**Results:**

The impact of BRV on memory is dose-dependent and mainly high doses may alter retrieval memory and fear-learning. Sub-chronic administration also impaired retrieval and spatial memory in animals. Moreover, chronic BRV may increase anxiety levels in rats but did not affect recognition memory.

**Conclusions:**

BRV may cause transient memory deficits as well as anxiety disturbances. However, the results are varied and depend on the type of memory, used dose, and duration of administration.

## Introduction

Brivaracetam (BRV), a molecular analog of levetiracetam (LEV), is one of the newest antiseizure medications. Compared to LEV, BRV selectively binds to synaptic vesicle glycoprotein 2A (SV2A) with approximately 20-fold higher affinity [1] and penetrates the blood-brain barrier more effectively [1, 2]. Despite their similar chemical structures, the drugs also differ in their effect on ion channels. Unlike LEV, BRV has an inhibitory effect on the sodium current; however, this effect does not seem to contribute to its antiseizure properties [3]. It has also been demonstrated that the drug has neuroprotective activity. BRV significantly reversed zinc modulation of GABA and glycine receptors at 10- to 30-fold lower concentrations than LEV [4]. Zinc-induced neurotoxicity is important in neuronal damage and death associated with traumatic brain injury, stroke, seizures, and neurodegenerative diseases [5]. Furthermore, BRV showed a protective activity against neuronal damage caused by status epilepticus in rats. The drug significantly increased the level of Bcl-2 proteins, which regulate the apoptosis process, and lowered the levels of apoptosis markers including annexin V and p53 [6].

Although brivaracetam is currently approved exclusively for adjunctive treatment of partial-onset seizures, it is currently under evaluation for its possible use in other types of epilepsy [7, 8]. Due to its interesting mechanism of action and favorable pharmacokinetic profile, the drug is also currently being reassessed with the aim of broadening its indications. In preclinical studies, the BRV has been evaluated for the treatment of migraine [9], neuropathic pain [10], and Alzheimer's disease [11]. In addition, its impact on memory disorder is also under investigation. It has been demonstrated that BRV may improve attention and executive functions in epilepsy patients. Patients also showed self-reported improvements in concentration and comprehension [12].

SV2A expression is ubiquitous in the brain and may play a significant role in the cognitive process. It has been demonstrated that a decrease in SV2A expression in the mouse hippocampus is associated with an increase in anxiety and spatial memory deficits [13]. These cognitive problems occur not only in patients with neurodegenerative diseases, like Alzheimer's disease but also in epileptic patients [14, 15]. It is known that patients with epilepsy have cognitive impairment, including memory, attention, and executive function deficits. These cognitive problems are caused by interlinked factors, including the pathophysiology of epilepsy and antiseizure treatment [16]. It has also been demonstrated that there is a bidirectional association between Alzheimer's disease and epilepsy [17, 18]. Moreover, anxiety may also occur in relation to seizures in epilepsy patients [19]. Most antiseizure medications can worsen the condition of the patient by impairing cognitive and behavioral functions [20]. Indeed, first-generation and some newer antiseizure drugs have been frequently associated with cognitive deterioration, contributing to a significant reduction in quality of life [21]. In contrast, brivaracetam is considered to be relatively safe and seems to be associated with fewer central adverse events. A meta-analysis encompassing 2505 patients found BRV to be well tolerated and rarely associated with serious adverse effects: the most commonly reported effects during BRV treatment were dizziness and fatigue [22]. Cognitive disturbance was generally not observed in preclinical studies [11, 23] or clinical studies [12]. However, some studies demonstrated rare cognitive deficits in patients treated with BRV [24].

Impairments in spatial memory and anxiety are noted in patients with epilepsy, reducing their quality of life [25]. To clarify the effects of brivaracetam on the central processes, the present study evaluates its influence on various types of memory and anxiety-like behaviors in rats. Spatial memory was assessed using the Morris water maze (MWM), the emotional memory associated with fear with the passive avoidance test (PA), and short- and long-term recognition memory with the novel object recognition test (NOR). Anxiety level was determined using the elevated plus maze test (EPM).

## Materials and methods

### Animals

80 adult Wistar rats (male, 252–273 g) were used in the experiment. The animals were bred in the Mossakowski Institute of Experimental and Clinical Medicine (Warsaw, Poland). The rats were housed in plastic cages in the number of four and maintained in a temperature-controlled room (20–22 °C) with a 12-h light/12-h dark cycle (7 a.m.–7 p.m. – light phase /7 p.m.–7 a.m. dark phase). All rats had free access to water and were provided the same food. The study was performed between 8:00 a.m. and 4:00 p.m.

The experiments were carried out according to the European Union Directive 2010/63/EU and Polish governmental regulations concerning experiments on animals (Dz.U.05.33.289) and approved by the Local Ethical Committee for Experimentation on Animals in Łódź (no. 9) (resolutions no. 66/ŁB/121/2018; 9/ŁB231/2022).

### Drug

**Fig. 1:**
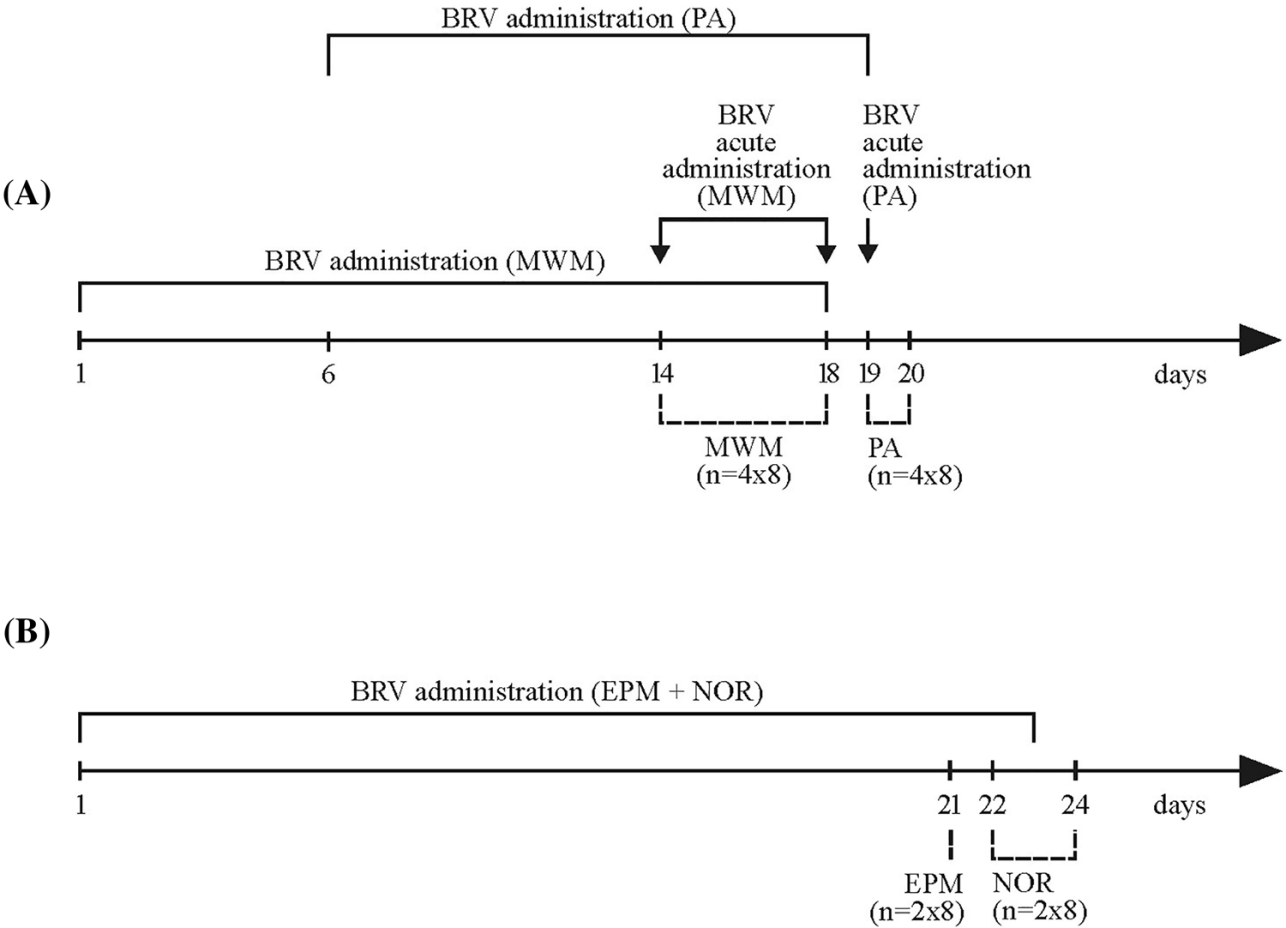
The timeline of experiment procedures. BRV – brivaracetam, MWM – Morris water maze test, PA – Passive avoidance test, EPM – Elevated plus maze test, NOR – Novel object recognition test. The dashed lines show the test performance. The solid lines show chronic administration of BRV and solid lines with vertical arrows demonstrate acute administration. The rats received an acute dose (6 mg/kg or 20 mg/kg *ig*) of BRV before the test and a chronic dose (6 mg/kg *ig*) after the end of the test

Brivaracetam as a ready-made solution (Briviact^®^, 10 mg/ml) was delivered intragastrically (*ig*) via an oral gavage. To investigate the acute effect of the BRV, a single dose of the drug (6 mg/kg or 20 mg/kg) was administered about 90 min before the trial. In a sub-chronic or chronic study, rats received BRV once a day (6 mg/kg) for 14 (MWM, PA) or 21 days (EPM, NOR). The scheme of the experiment is shown in Fig. 1. Separate groups were involved in the MWM test (BRV_H(MWM), BRV_L(MWM), BRV_14(MWM), CNT(MWM)), PA test (BRV_H(PA), BRV_L(PA), BRV_14(PA), CNT(PA)) and NOR/EPM tests (BRV_21(EPM+NOR), CNT(EPM+NOR)). On test days, BRV was given after behavioral tests to avoid the influence of the acute dose on the obtained results. The 1% aqueous solution of methylcellulose (0.2 ml/100 g) was administered to the control groups (CNT(MWM), CNT(PA), CNT(EPM+NOR)) for 14 (MWM, PA) or 21 days (NOR, EPM). In the MWM and PA tests, there was a common control group for acute and sub-chronic dose studies, which is a limitation of our studies. However, we decided to reduce the number of animals according to the 3R rule due to our previous experience that rats swim and enter the dark compartment very similarly, regardless of whether they receive a placebo in a single dose or multiple doses.

### Morris water maze test (MWM)

The MWM is a common behavioral test assessing spatial learning and memory. A correct learning process is indicated by a shorter search time and swimming distance, and a longer time in the zone with platform in the following test days. The test was performed in a circular pool with a diameter of 180 cm and a depth of 50 cm. The tank was filled with water (23±1 °C) and several spatial cues were placed on the interior of the pool to facilitate navigation by the rat. The pool had four virtual sections and a hidden platform with a diameter of 8 cm which was placed in the center of the selected quadrant about 2 cm below the surface of the water. MWM was performed in a quiet room and the animal had no eye contact with the researcher during the tests. All experiments were recorded with a camera hanging above the pool. ANY-maze software (ANY-maze, USA) was used to track the movement of the rodent and the entrance to the platform.

The five-day MWM scheme was chosen [26]. The test started with acquisition tests which were performed over the four following days. The platform was positioned in the same place each time. The rat started each 60-s trial in a selected quadrant (different start quadrant each day) and was put in the pool facing the wall. The scheme of putting animals in the pool was the same for all animals each day. If the rat found the platform in the allotted time, it stayed on it for 15 s to remember the environment. If the trail was unsuccessful, the rat was moved there manually by the researcher and also allowed to remain there for 15 s. Four trials were conducted during each daily session for each animal with a 60-s interval between them. The decrease in time latency to locate the platform reflects functional spatial memory and learning. A probe trial without a hidden platform was carried out on the fifth day. The animals were allowed to swim freely for 60 s; if the retrieval memory was undisturbed, the rodents should primarily stay in the quadrant that previously contained the platform.

### Passive avoidance test (PA)

The passive avoidance test (PA) is intended to assess fear learning in rodents [27]. During the experiment, the rats learn to avoid a preferred dark compartment with an aversive stimulus. The step-through PA was performed in a two-chamber apparatus with a guillotine door (Gemini Avoidance System, San Diego, USA). Each chamber had dimensions of 25 mm (width) × 20 mm (depth) × 17 mm (height).

The experimental procedure consisted of two trials separated by a 24 h interval. The first day of the test was an acquisition trial in which a single test was carried out. Firstly, each rat was placed in the dark compartment. After a 60-s habituation period, the light was turned on and the door was raised automatically. The rat could move to the dark compartment, which is the animal’s natural preference. As soon as the rat placed all four paws in the dark compartment, the gate was closed and a foot shock (0.5 mA, 3 s) was delivered through the floor grid. The rat remained in the dark compartment for 15 s to associate an aversive stimulus (foot shock) with the environment. 70% isopropyl alcohol was used to clean the apparatus with between trials.

The retention trial was carried out 24 h after the acquisition trial. Each rat was placed in the light compartment, and the trail ended when the rodent entered the dark compartment or when it remained in the light compartment for 300 s. No electric shock was given to the rats in this trail. The measured parameter was the step-through latency to the dark compartment. A longer step-through latency indicates an undisturbed memory of the aversive stimulus.

### Novel object recognition test (NOR)

The recognition memory was assess using NOR test. Three phases comprise the test: habituation, familiarization, and testing [28]. The habituation phase was carried out on the first test day: the rat was placed into an empty plastic box and was allowed to become acquainted with the environment freely for 5 min. The next day, the rat entered the familiarization phase, during which it was put into the same box but with two identical objects and allowed to explore them for 3 min. The short-term memory was assessed after a 5-min break: the animal was presented with the same 3-min trial but with one novel and one familiar object. The long-term memory was assessed during a similar trial after 24 h. The rats were exposed to two objects, one of which was again replaced with a new object and the other object was familiar. Exploratory behavior was considered as directing the nose toward the object at a distance no further away than 1 cm or touching it with the nose. Turning around or sitting on the object was not considered as exploratory behaviors. To maintain the same fragrance, 70% isopropyl alcohol was used to clean the box and objects. Memory was expressed by the discrimination index (DI), defined as the formula: novel object (s)/novel object (s) + familiar object (s) × 100%. A DI value higher than 50% reflects a preference for the novel object, while a value lower than 50% indicates a preference for the familiar object. A score of around 50% means no preference.

### Elevated plus maze test (EPM)

The elevated plus maze test (EPM) was used to assess anxiety-like behavior and was conducted as described previously [28]. The EPM apparatus consisted of four arms; two of them were open and two were closed. The arms were 10 cm wide, 50 cm long, and were placed about 50 cm above the floor. A start platform (5 cm × 5 cm) was located in the center of maze. At the beginning of the trail, the rat was placed in the center of the apparatus facing an open arm. The animal was allowed to explore freely the apparatus for 5 min, and its behavior was recorded with a camera mounted above the maze. The following behavioral parameters were automatically determined: the percentage of time in the open arms (time in open arms/total time in the arms), the percentage of open arm entries (open arm entries/total arm entries), and the distance traveled. All four paws must be placed in the arm to confirm “entry” to the selected arm. The apparatus was cleaned with 70% isopropyl alcohol between trails to maintain the same fragrance.

### Statistical analyses

Statistica 13.3 software was used to analyze the results were analyzed. The non-parametric tests were chosen, as the assumptions of the normal distribution (Kolmogorov-Smirnov test with Lilliefors correction) and homogeneity of variances (Levene’s test) for a parametric test were not fulfilled. In the MWM, the Kruskal-Wallis test was used to compare groups on a particular day, with the Friedman *post hoc* test to compare within a single group. The Kruskal-Wallis was also used for comparing one parameter in the EPM (*viz.* percent entries) and for between-group comparisons in the PA. The Student’s *t*-test was employed to examine the rest of the parameters in the EPM and recognition memory in the NOR. For all statistical tests, a *p*-value of 0.05 or less indicated a statistically significant difference. Data are present as mean values ± SD (parametric tests) or median (horizontal bar), first and third quartiles (vertical column), and minimum and maximum (vertical line) (non-parametric tests). Outlier values are presented with circles. Individual data points have been marked with black triangles.

## Results

### The effect of brivaracetam administered at a single low (6 mg/kg *ig*) or high dose (20 mg/kg *ig*) on spatial memory in rats in MWM

**Fig. 2:**
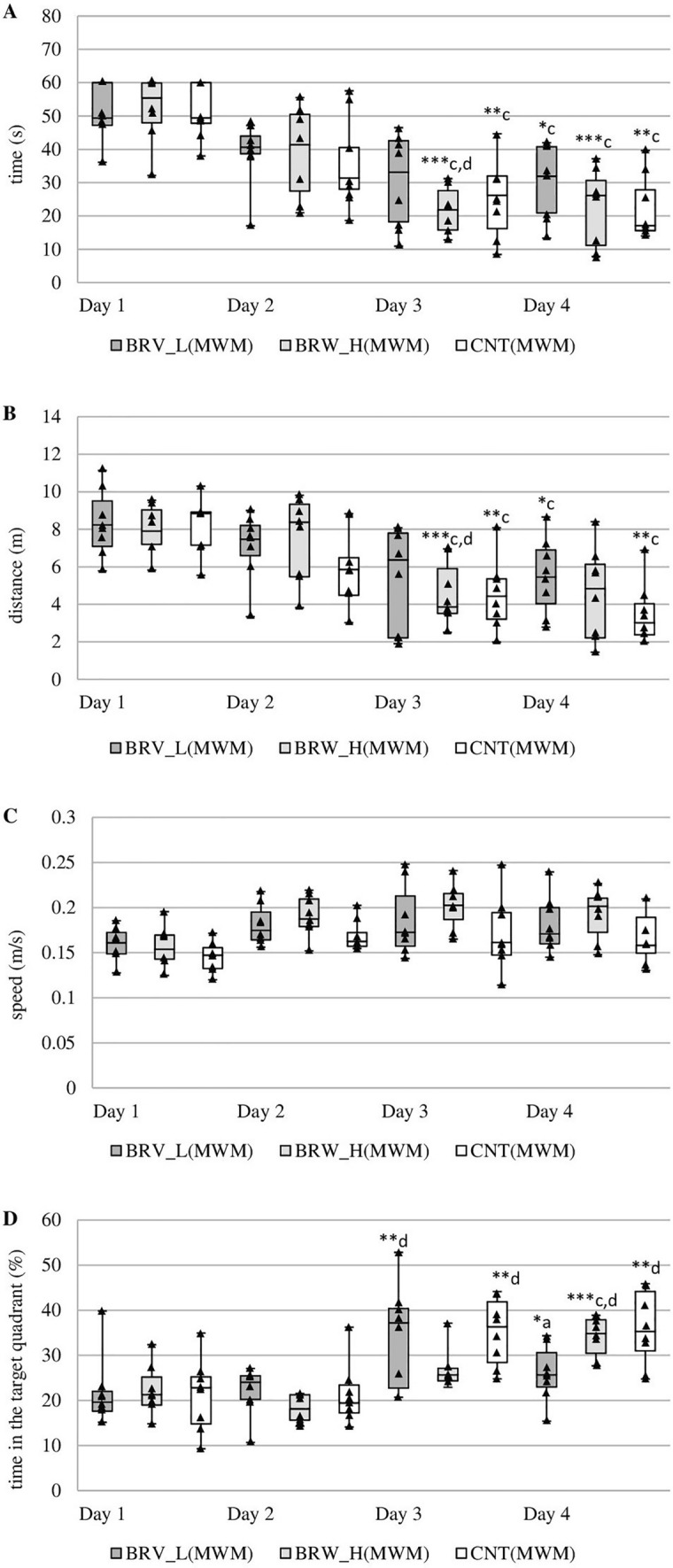
Effect of acute administration of low and high doses of brivaracetam (BRV) in the Morris water maze (MWM) on the time needed to localize the platform (**A**), the distance traveled by rats to localize the platform (**B**), swimming speed (**C**), the time spent in the zone with the platform (**D**); BRV_L(MWM) – group received a low dose (L) of BRV (6 mg/kg *ig*) (*n* = 8), BRV_H(MWM) – group received a high dose (H) of BRV (20 mg/kg *ig*) (*n* = 8), CNT(MWM) – control group (*n* = 8); ^a^Statistically significant difference between BRV_L(MWM) and CNT(MWM) groups on that day; Kruskal-Wallis test. ^c^ Statistically significant difference between a particular test day and test day 1; the Friedman test. ^d^Statistically significant difference between particular test day and test day 2; the Friedman test; *p < 0.05, **p < 0.01, ***p < 0.001. The data are presented as box plots, with the horizontal line indicating the median, and vertical boxes and whiskers depicting the percentile range

BRV administered at a dose of 6 mg/kg or 20 mg/kg did not affect the time needed to find the platform (Fig. 2A). This time was also shortened throughout the test for all groups of animals. Significant differences were observed between the initial values and those on the fourth day for BRV_L(MWM) (Chi sq = 9.45, *N* = 8, *p* = 0.024, Friedman test), BRV_H(MWM) (Chi sq = 18.15, *N* = 8, *p* = 0.001, Friedman test) and CNT(MWM) (Chi sq = 14.55, *N* = 8, *p* = 0.002, Friedman test). On day 3, the BRV_H(MWM) group needed significantly less time to find the platform compared to days 1 and 2 (Chi sq = 18.15, *N* = 8, *p* = 0.001, Friedman test). Similar reductions in time were noted for the CNT(MWM), with significant differences observed on day 3 compared to initial values (Chi sq = 14.55, *N* = 8, *p* = 0.002, Friedman test).

The rats from all groups swam comparable distances on the following days (Fig. 2B). The BRV-treated rats (BRV_L(MWM) and BRV_H(MWM)) demonstrated an increased distance on days 2 and 4, but these differences were not statistically significant. The distance gradually shortened over time, and significant differences were found between the initial values and day 4 in BRV_L(MWM) (Chi sq = 8.25, *N* = 8, *p* = 0.041, Friedman test) and CNT(MWM) (Chi sq = 14.55, *N* = 8, *p* = 0.002, Friedman test). A similar reduction was also observed on day 3 compared to the initial values for CNT(MWM) (Chi sq = 14.55, *N* = 8, *p* = 0.002, Friedman test). On day 3, the BRV_H(MWM) group also needed significantly less time to find the platform compared to days 1 and 2 (Chi sq = 18.15, *N* = 8, *p* = 0.001, Friedman test). As shown in Fig. 2C, the low-dose drug did not significantly affect swimming speed. During the four days of the MWM test, the high-dose group (BRV_H(MWM)) demonstrated a higher swimming speed but these differences were not significant. In contrast, on day 4, low-dose brivaracetam (BRV_L(MWM)) significantly decreased the time the rats spent in the zone with the platform compared to the CNT(MWM) group (H(2, N = 24) = 6.66, *p* = 0.036, Kruskal-Wallis test) (Fig. 2D). On the other hand, these animals demonstrated a significantly longer time in the proper zone on day 3 compared to baseline (Chi sq = 11.55, *N* = 8, *p* = 0.009, Friedman test). High-dose BRV (BRV_H(MWM)) significantly lengthened the time in the proper zone on the last day compared to days 1 and 2 (Chi sq = 17.70, *N *= 8, *p* = 0.001, Friedman test). In the control group, the time spent in the proper zone was significantly higher on days 3 and 4 compared to day 2 (Chi sq = 14.85, *N* = 8, *p* = 0.002, Friedman test).

**Fig. 3:**
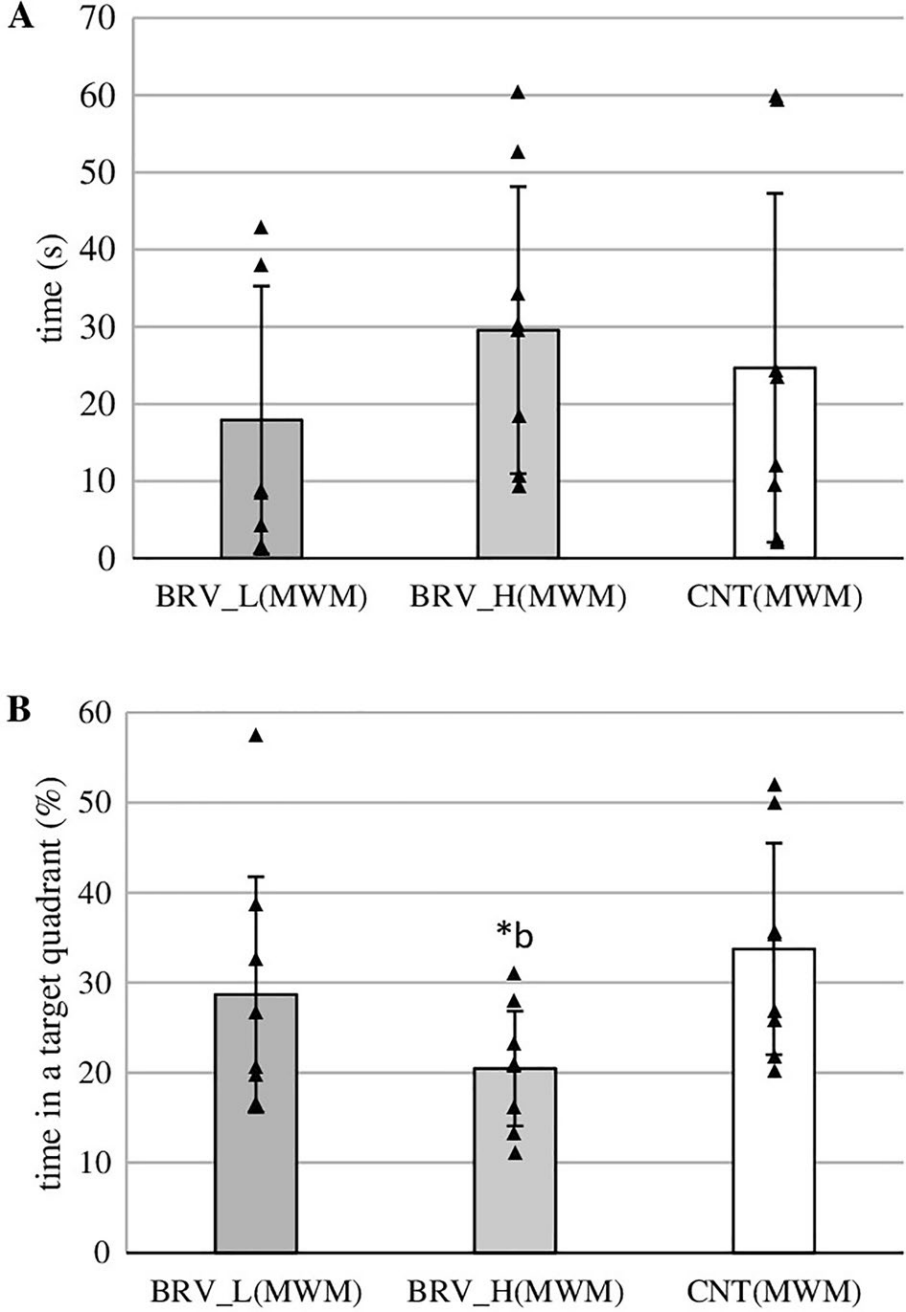
Effect of acute administration of low and high doses of brivaracetam (BRV) on the fifth day of the Morris water maze (MWM) (without platform) on the time needed to identify the previous location of the platform (**A**), the time spent in the zone where the platform was located previously (**B**); BRV_L(MWM) – group received a low dose (L) of BRV (6 mg/kg *ig*) (*n* = 8), BRV_H(MWM) – group received a high dose (H) of BRV (20 mg/kg *ig*) (*n* = 8), CNT(MWM) – control group (*n* = 8); ^b^ Statistically significant difference between BRV_H(MWM) and CNT(MWM) groups on that day; Student’s *t*-test, * p < 0.05. Data are presented as mean values ± SD

On the fifth day, when the platform was removed, the rats receiving low-dose BRV (BRV_L(MWM)) would find the platform in slightly less time compared to CNT(MWM) and BRV_H(MWM) but the differences were not statistically significant (Fig. 3A). As noted in Fig. 3B, the rats receiving high-dose BRV (BRV_H(MWM)) spent a significantly lower percentage of time in the zone where the platform had been located than CNT(MWM) (*t *= 10.302, *p* = 0.034, Student’s *t*-test). No significant difference was found between BRV_L(MWM) and CNT(MWM) or BRV_L(MWM) and BRV_H(MWM) with regard to the percentage of time spent in the correct zone.

### The effect of brivaracetam administered sub-chronically on spatial memory in rats in MWM

**Fig. 4:**
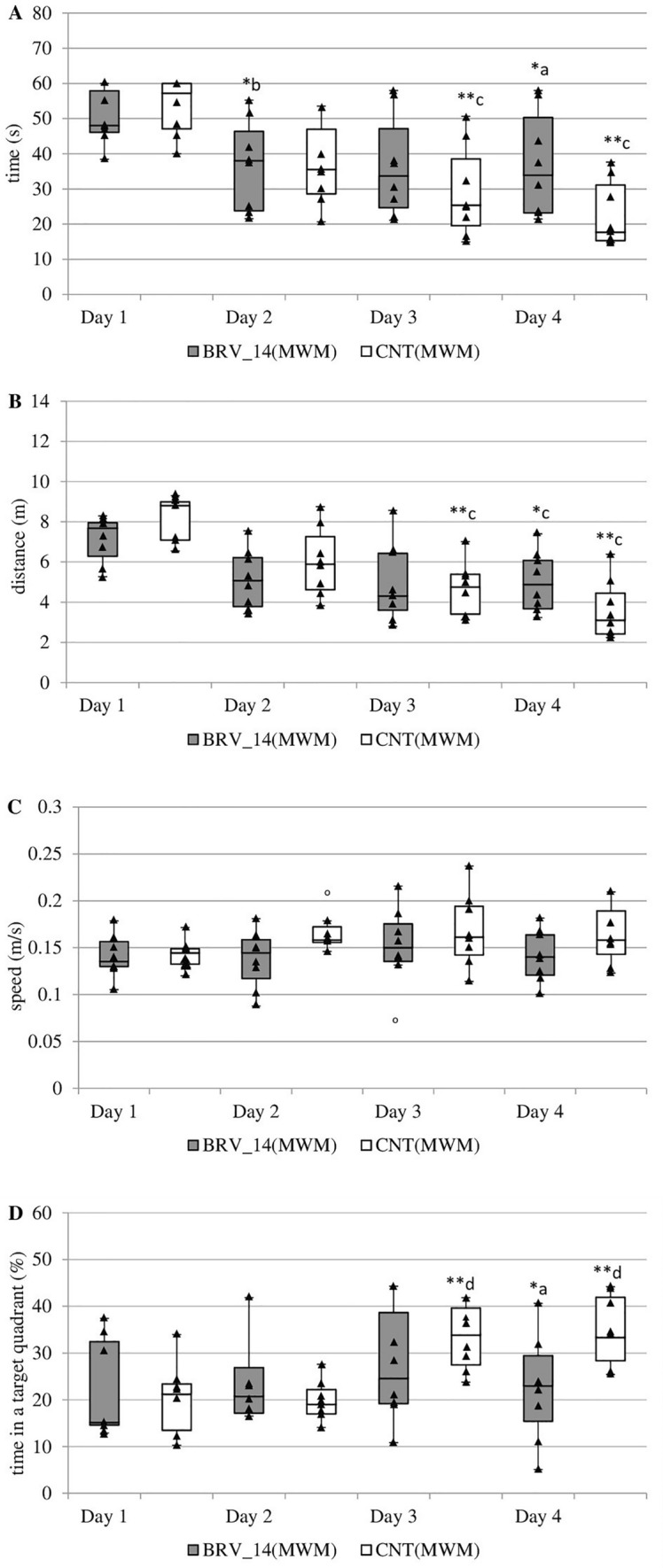
Effect of sub-chronic administration of brivaracetam (BRV) in the Morris water maze (MWM) on the time needed to localize the platform (**A**), the distance traveled by rats to localize the platform (**B**), swimming speed (**C**), the time spent in the zone with the platform (**D**); BRV_14(MWM) – group received BRV for 14 days (6 mg/kg *ig*) (*n* = 8), CNT(MWM) – control group (*n* = 8); ^a^Statistically significant difference between BRV_14(MWM) and CNT(MWM) groups on that day; Kruskal-Wallis test ^c^Statistically significant difference between particular test day and test day 1; Friedman test ^d^Statistically significant difference between particular test day and test day 2; Friedman test; *p < 0.05, **p < 0.01. The data are presented as box plots, with the horizontal line indicating the median, and vertical boxes and whiskers depicting the percentile range

BRV administered for 14 days at a dose of 6 mg/kg significantly increased the time needed to find the platform on day 4 (H(1, N = 16) = 4.41, *p* = 0.036, Kruskal-Wallis test) (Fig. 4A). This time was initially reduced and a significant difference was observed between days 1 and 2 (Chi sq = 10.30, *N *= 8, *p* = 0.016, Friedman test). In the control group, a significant decrease of this time was noted on days 3 and 4 compared to the initial values (Chi sq = 14.55, *N* = 8, *p* = 0.002, Friedman test).

BRV administration did not result in any changes in the distance traveled (Fig. 4B) and a significant reduction in distance was observed between days 1 and 4 in both groups (Chi sq = 8.55, *N* = 8, *p* = 0.036, Friedman test (BRV_14(MWM)); Chi sq = 14.55, *N* = 8, *p* = 0.002, Friedman test (CNT(MWM))) and between days 1 and 3 in the CNT(MWM) group (Chi sq = 14.55, *N* = 8, *p* = 0.002, Friedman test). Furthermore, the BRV rats demonstrated a slower swimming speed on day 4 than the controls, but no significant differences were noted (Fig. 4C). The BRV_14(MWM) group spent less time in the zone with the platform than CNT(MWM) on day 4 (H(1, N = 16) = 4.41, *p* = 0.036, Kruskal-Wallis test) (Fig. 4D). On all days, the tested parameter remained statistically unchanged in the BRV_14(MWM) group, but was prolonged in the CNT(MWM) group. Significant differences were observed between days 3 and 4 compared to day 2 (Chi sq = 14.85, *N* = 8, *p* = 0.002, Friedman test).

**Fig. 5:**
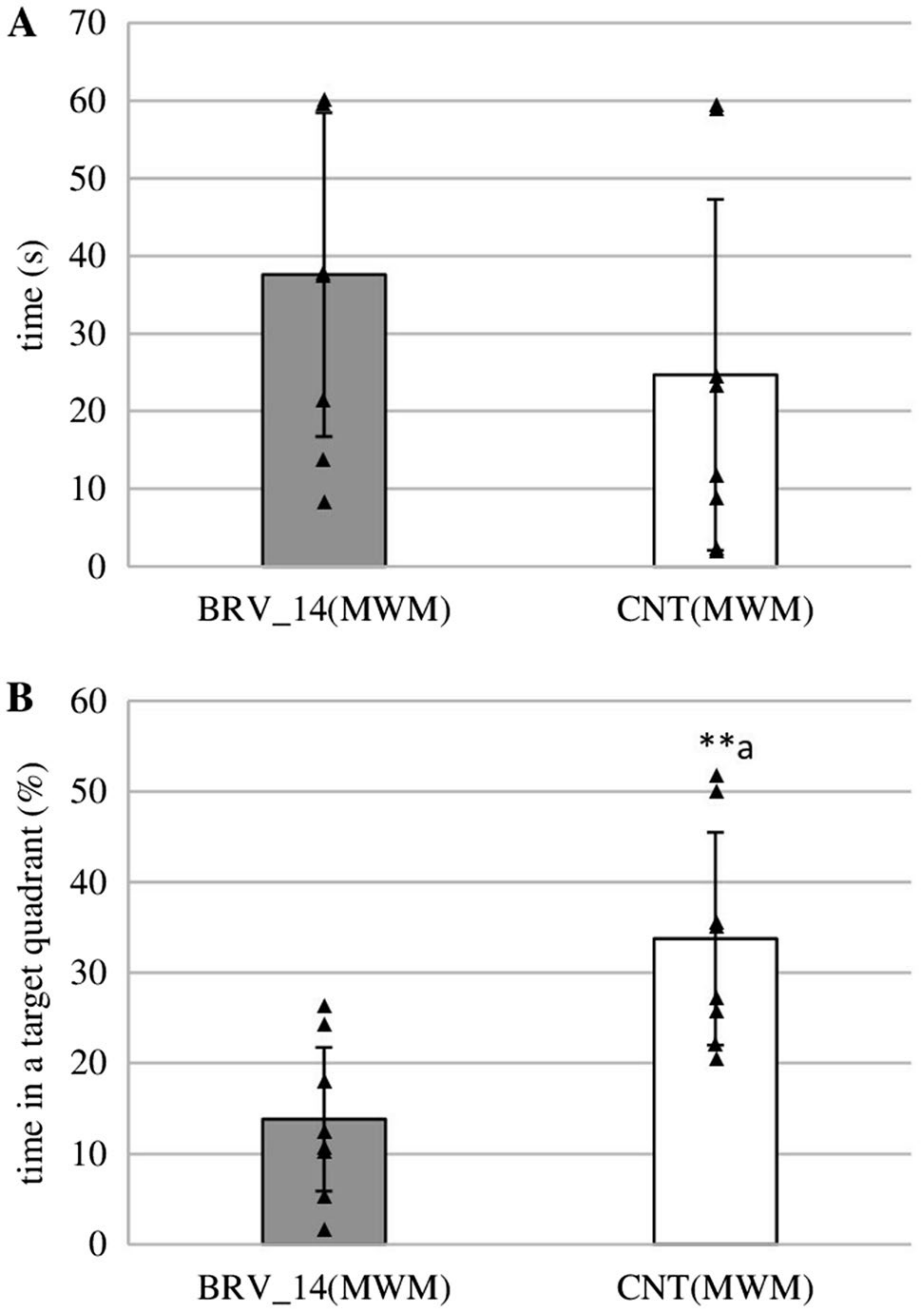
Effect of sub-chronic administration of brivaracetam (BRV) on the fifth day of the Morris water maze (MWM) (without platform) on the time needed to identify the previous location of the platform (**A**), the time spent in the zone where the platform was located previously (**B**); BRV_14(MWM) – group received BRV for 14 days (6 mg/kg *ig*) (*n* = 8), CNT(MWM) – control group (*n* = 8); ^a^Statistically significant difference between BRV_14(MWM) and CNT(MWM) groups on that day; Student’s *t*-test, **p < 0.01. Data are present as mean values ± SD

On the last day of the MWM test, the time to find the virtual platform was slightly lower in the control group but this difference was not significant (Fig. 5A). However, rats receiving BRV for 14 days spent significantly less time in the zone where the platform was previously located than controls (*t* = −3.976, *p* = 0.001, Student’s *t*-test) (Fig. 5B).

### The effect of brivaracetam administered at a single acute dose or sub-chronically on emotional memory in rats in PA

**Fig. 6:**
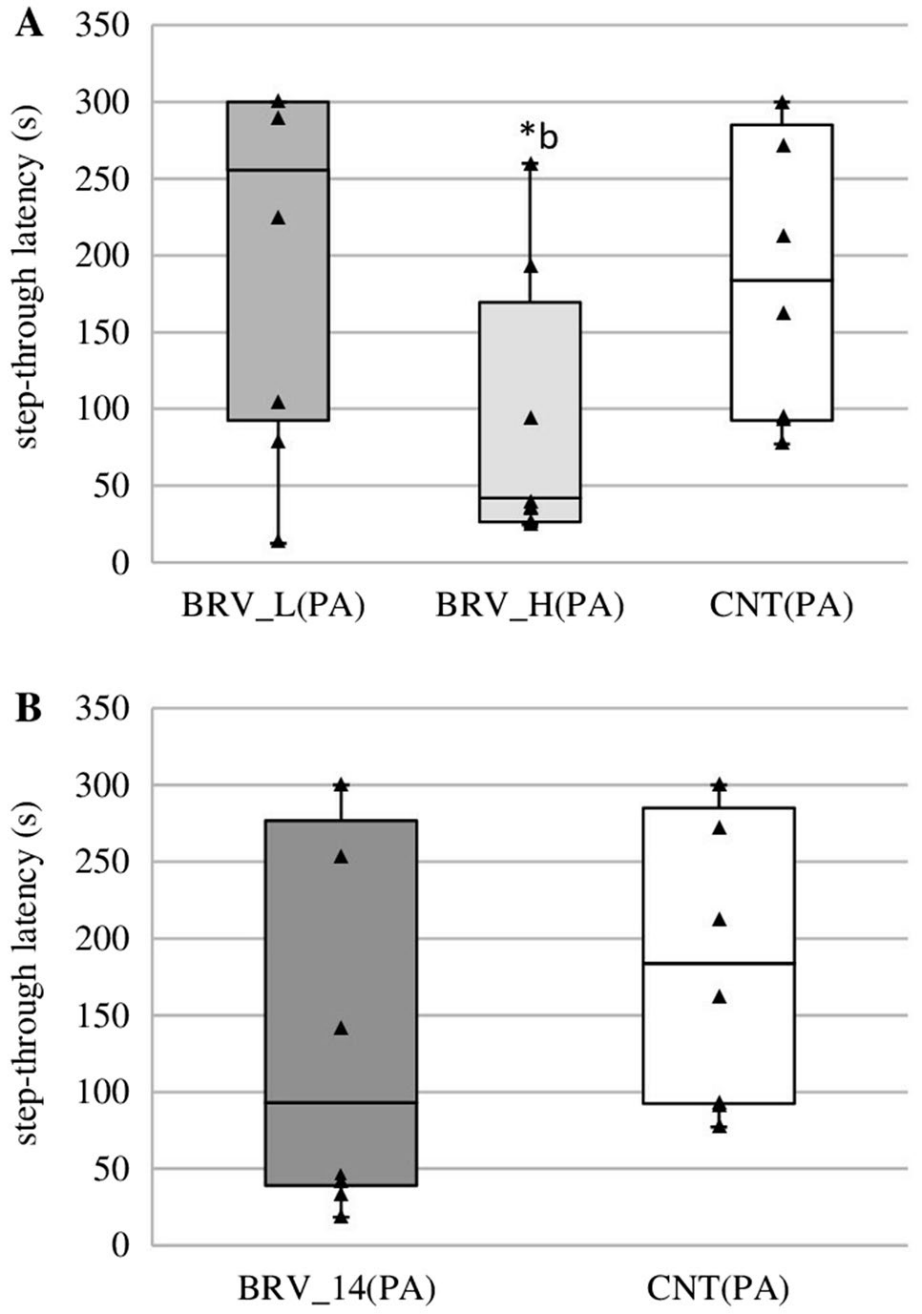
Effect of low dose and high dose (**A**), and sub-chronic administration (**B**) of brivaracetam (BRV) on fear learning in the passive avoidance (PA). BRV_L(PA) – group received a low dose (L) of BRV (6 mg/kg *ig*) (*n* = 8), BRV_H(PA) – group received a high dose (H) of BRV (20 mg/kg *ig*) (*n* = 8), BRV_14(PA) – group received BRV for 14 days (6 mg/kg *ig*) (*n* = 8), CNT(PA) – control group (*n* = 8);^b^ Statistically significant difference between BRV_H(PA) and CNT(PA) groups on that day; Kruskal-Wallis test, *p < 0.05. The data are presented as box plots, with the horizontal line indicating the median, and vertical boxes and whiskers depicting the percentile range

The single low dose of BRV (6 mg/kg) did not alter the step-through latency to enter the dark compartment (Fig. 6A); however, the single high dose (20 mg/kg) caused a significant decrease in comparison to CNT(PA) (H(2, N = 24) = 6.073, *p* = 0.048, Kruskal-Wallis test) (Fig. 6A). Similarly to the low dose, BRV administered for 14 days (6 mg/kg) did not affect the step-through latency value (Fig. 6B).

### The effect of brivaracetam administered chronically on short- and long-term memory in NOR

**Fig. 7:**
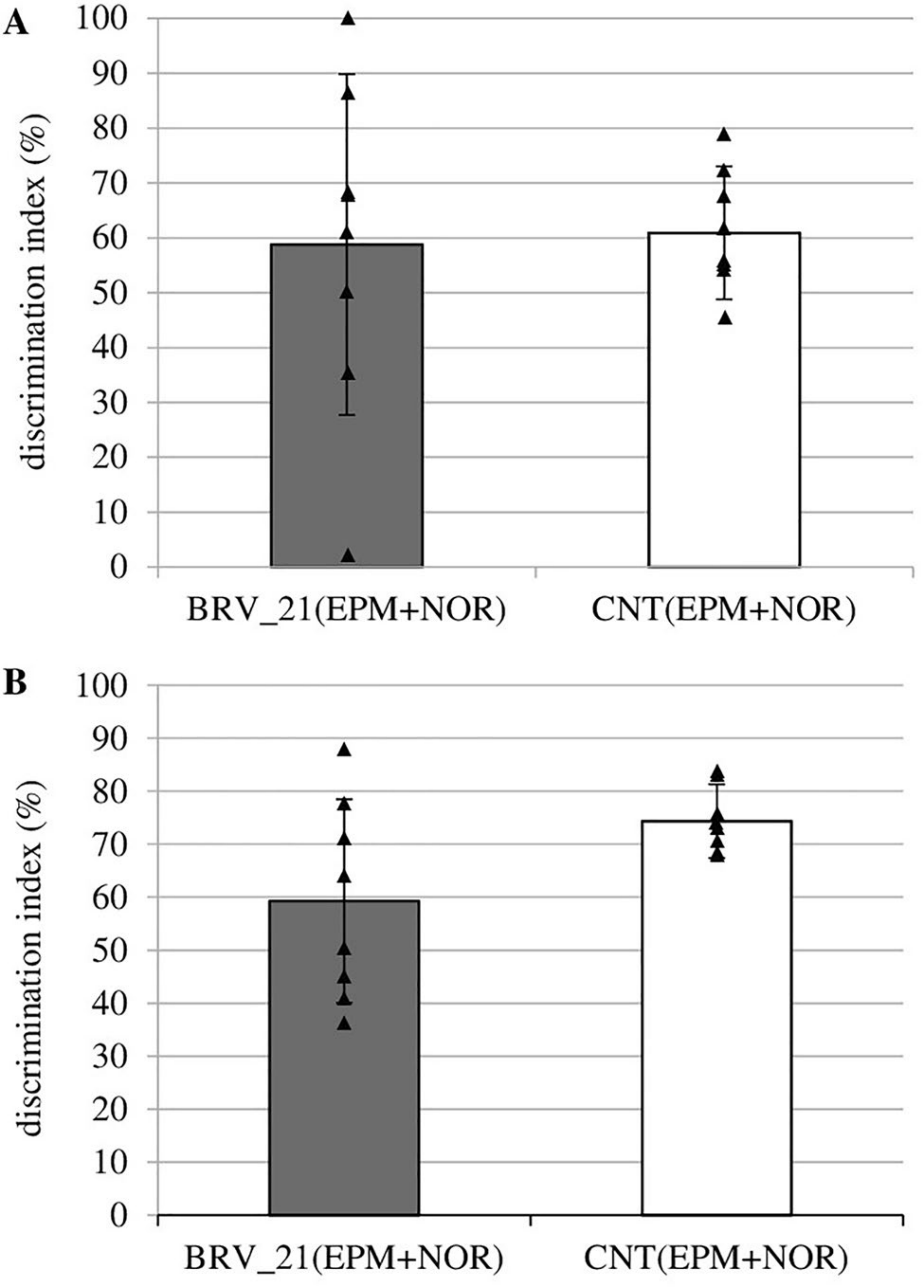
Effect of chronic administration of brivaracetam (BRV) on short-term (**A**) and long-term memory (**B**) in the novel object recognition (NOR). Student’s *t*-test. BRV_21(EPM+NOR) – group received BRV for 21 days (6 mg/kg *ig*) (*n* = 8), CNT(EPM+NOR) – control group (*n* = 8); Data are present as mean values ± SD

BRV administered for three weeks did not affect the discrimination index of short-term memory (Fig. 7A). The DI value was slightly decreased in the BRV_21(EPM+NOR) group, but no significant difference was observed in comparison with the control group values (Fig. 7B).

### The effect of brivaracetam administered chronically on anxiety-like behaviors in EPM

**Fig. 8:**
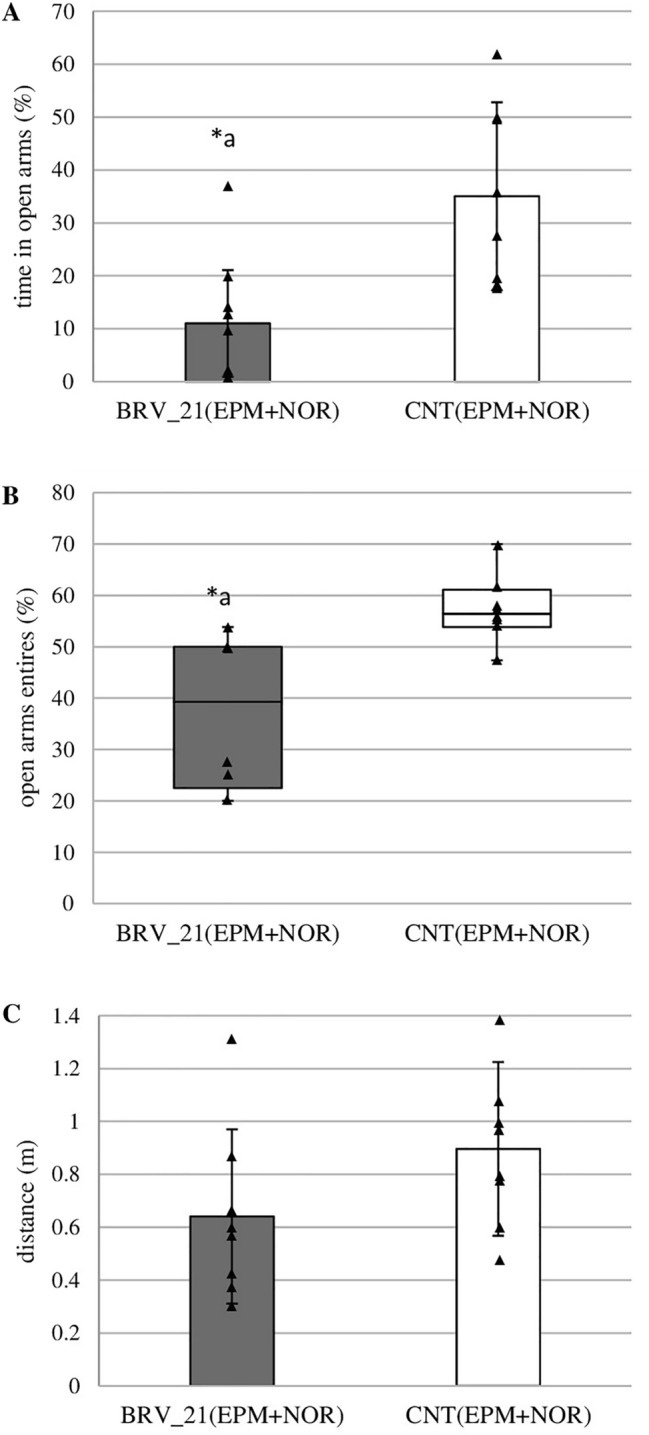
Effect of chronic administration of brivaracetam (BRV) on time in the open arms (**A**), open arms entries (**B**), and traveled distance (**C**) in the elevated plus maze (EPM). BRV_21(EPM+NOR) – group received BRV for 21 days (6 mg/kg *ig*) (*n* = 8), CNT(EPM+NOR) – control group (*n* = 8); ^a^Statistically significant difference between BRV_21(EPM+NOR) and CNT(EPM+NOR) groups on that day; Student’s *t*-test (**A**) or Kruskal-Wallis test (**B**), *p < 0.05. Data are present as mean values ± SD (**A**, **C**) or as box plots, with the horizontal line indicating the median, and vertical boxes and whiskers depicting the percentile range (**B**)

BRV administered for three weeks decreased the percentage of the time in open arms (*t* = 2.946, *p* = 0.012, Student’s *t*-test) (Fig. 8A) and the percentage of open arms entries (H(1, *N* = 16) = 6.42, *p* = 0.011, Kruskal-Wallis test) (Fig. 8B). Although the traveled distance was shorter in the BRV_21(EPM+NOR) group, no significant difference was observed between groups (Fig. 8C).

## Discussion

The obtained results suggest that BRV may affect spatial and retrieval memory. However, this effect appears to be dependent on the dose or duration of BRV administration because only sub-chronic BRV impaired spatial memory on day 4 of the MWM. A single low dose of BRV also disturbed one of the tested parameters of spatial memory which may indicate transient cognitive impairment. Moreover, a high dose and sub-chronic BRV can alter retrieval memory tested on the last day of the MWM. BRV impaired also fear learning in the PA but this effect was only observed in rats receiving a single high dose of BRV. Furthermore, chronic BRV increased anxiety in the EPM but did not affect recognition memory in the NOR. Observed central disturbances may be key factors limiting its possible use.

Our findings indicate that BRV administered at a single high dose did not impair spatial memory in rats: the time needed to find the platform and the traveled distance was shortened throughout the test. A single low dose of BRV (6 mg/kg) caused a reduction in time spent in the zone with platform on day 4 of the test compared to the control group. This did not significantly affect the time to find the platform, but it may indicate the appearance of transient cognitive impairment, which might be related to the accumulation of the drug. Data from healthy subjects indicate that the drug and its metabolites do not tend to accumulate after oral administration and the half-life of BRV is between 7 and 8 h. However, the study was conducted only on six men receiving a single dose of BRV [29]. Memory impairment was also observed on day 4 of the study in rats receiving BRV for 14 days. These animals needed significantly more time to find the platform and spent less time in the zone with the platform. These disorders are indicative of BRV-induced impairment of spatial memory and may be also related to the accumulation of the drug. However, there are no available data on the accumulation rate after long-term administration.

In contrast, Detrait et al. (2010) suggested that BRV has no adverse effect on hippocampal-dependent cognitive functions. In MWM, the drug did not impair spatial memory in either normal or amygdala-kindled rats after a single dose of BRV (2.1, 6.8, 21 mg/kg *ip*). In addition, the authors observed that BRV did not affect LTP induction in the CA1 region of the hippocampus [30]. In another study, BRV fully reversed spatial memory impairments in transgenic mice with Alzheimer’s disease [11]. As mentioned previously, BRV selectively binds to SV2A [1] and an increase in anxiety and spatial memory deficits is associated with a hippocampal decrease in SV2A expression [13]. There is no data available in the literature on the effect of BRV in animals with under-expression of SV2A. However, it has been demonstrated that BRV ameliorated the over-expression of SV2A in the hippocampus of epileptic rats. An increased level of SV2A was associated with depressed long-term potentiation in the hippocampus and the drug reversed the synaptic dysfunction [31].

Our present study also demonstrated that brivaracetam has a low impact on swimming speed. While a single low dose appeared to have no effect on swimming speed, a high dose tended to increase swimming speed compared to controls. In contrast, animals receiving BRV chronically swam slowly; however, none of these differences were significant. Detrait et al. (2010) found BRV to have no effect on swimming speed, regardless of the dose [30]. No effect of BRV on locomotion was also observed in open field test [23].

The retrieval memory was assessed on the last day of the MWM study. Neither low nor high single doses of BRV were found to affect significantly the potential time needed to find the platform. However, the animals receiving high or sub-chronic doses spent significantly less time in the quadrant where the platform was previously located. In sub-chronic treated animals, this decrease correlated with a longer potential time to find the platform, although this difference was not significant. The decrease of the time spent in the target zone suggests the impairment of retrieval memory. A similar adverse effect was observed in these animals on the last day of acquisition tests which may indicate a permanent impairment.

Similar effects were observed by Sanon et al. (2018) who assessed the safety profile of BRV, including its effect on hippocampal-dependent memory. Male rats received BRV at a single dose of 30 mg/kg *ip*, and status epilepticus was induced in some rodents. On the last day of the Morris test, the BRV rats spent less time in the zone where the platform was previously located. This effect was seen in both epileptic and sham rats [23].

The second test used in the present study to assess recognition memory was the NOR test. The rats receiving 6 mg/kg BRV administered for three weeks did not demonstrate any significant difference in short-term or long-term recognition memory compared to controls. A lower discrimination index, indicating less recognition of novel objects, was noted in the long-term memory test, but again this was not a significant difference compared to controls.

Although no study has used the NOR test to study the effect of BRV on memory, previous clinical observations indicate that it has a negligible or even beneficial influence on cognitive functions. Witt et al. (2018) also found BRV used in add-on therapy to have a beneficial effect on executive functions and attention in epilepsy patients; however, verbal memory was unaffected. An improvement was also observed in spatial memory and orientation [12]. Similarly, BRV has been found to have beneficial effects on cognitive functions in healthy people and was well tolerated from a neuropsychological perspective [32].

The present study also used the Passive Avoidance test to evaluate fear learning and memory. The test, a fear-aggravated task, is based on the acquisition, storage, and maintenance of an aversive stimulus in memory. A dose-dependent disturbance was observed, manifested as the rats receiving BRV at a single high dose demonstrating a reduction in step-through latency; this indicates that the drug has a negative effect on memory, and reflects an impaired association of a dark compartment with an aversive stimulus. On the other hand, BRV administered at a single low dose or repeatedly for 14 days did not significantly alter cognitive functions.

In the available literature, there are no studies on the effect of BRV on memory associated with fear assessed in the PA test. Only Sanon et al. (2018) evaluated the impact of BRV at a single dose on fear learning in a fear-conditioning test. This test is associated with the exposure of the rodent to an aversive stimulus. Briefly, in the test, re-exposure to an unpleasant environment or sound stimulus resulted in an anxiety response, in this case, animal immobility, i.e. *freezing*, the duration of which indicates the degree of association between the unpleasant stimulus and the surrounding environment. BRV did not affect fear learning measured in this test [23].

The final task was to investigate the effect of BRV on anxiety levels in rats using the EPM test. Repeated administration of BRV significantly decreased the percentage of the time spent in the open arms, and the percentage of open arms entries, which is indicative of increased anxiety. Another preclinical study investigated the effect of BRV on anxiety levels in an EPM test in rats with status epilepticus induced by kainic acid. BRV administered in a single dose of 30 mg/kg *ip* did not affect anxiety levels [23]. The long-term safety of BRV was also evaluated in an 11-year, open-label, follow-up trial, with adverse psychiatric events assessed by the Hospital Anxiety and Depression Scale (HADS) for the first two years. One of the most common side effects was anxiety, which was reported in 45 patients (6.7%) [33].

All of the effects of BRV observed in this study are important from the clinical point of view, especially in long-term therapy or in patients with anxiety disorders. Considering these adverse effects, there is clearly a need for further studies to confirm the safety profile of BRV.

## Data Availability

The datasets generated during and/or analyzed during the current study are available from the corresponding author upon reasonable request.

## Abbreviations

BRV: Brivaracetam
BRV_14(MWM): The group received BRV for 14 days (6 mg/kg *ig*) in the Morris water maze
BRV_14(PA): The group received BRV for 14 days (6 mg/kg *ig*) in the passive avoidance
BRV_21(EPM+NOR): The group received BRV for 21 days (6 mg/kg *ig*) in the elevated plus maze and novel object recognition
BRV_H(MWM): The group received a high dose of brivaracetam (20 mg/kg *ig*) in the Morris water maze
BRV_H(PA): The group received a high dose of BRV (20 mg/kg *ig*) in the passive avoidance
BRV_L(MWM): The group received a low dose of brivaracetam (6 mg/kg *ig*) in the Morris water maze
BRV_L(PA): The group received a low dose of brivaracetam (6 mg/kg *ig*) in the passive avoidance
CNT(EPM+NOR): The control group in the elevated plus maze and novel object recognition
CNT(MWM): The control group in the Morris water maze
CNT(PA): The control group in the passive avoidance
EPM: Elevated plus maze
HADS: Hospital Anxiety and Depression Scale
*ig*: Intragastrical
*ip*: Intraperitoneal
LEV: Levetiracetam
MWM: Morris water maze
NOR: Novel object recognition
SV2A: Synaptic vesicle glycoprotein 2A
PA: Passive avoidance
